# Effects of Vocabulary and Phonotactic Probability on 2-Year-Olds’ Nonword Repetition

**DOI:** 10.1007/s10936-016-9448-9

**Published:** 2016-09-09

**Authors:** Josje Verhagen, Elise de Bree, Hanna Mulder, Paul Leseman

**Affiliations:** 1 Utrecht University, P.O. Box 80140, 3508 TC Utrecht, The Netherlands; 2 University of Amsterdam, Amsterdam, The Netherlands

**Keywords:** Nonword repetition, Vocabulary, Phonotactic probability, 2-Year-olds, Phonological representations

## Abstract

This study investigates the relationship between nonword repetition (NWR) and vocabulary in 2-year-olds. Questions addressed are whether (1) NWR and vocabulary are associated, (2) phonotactic probability affects NWR, and (3) there is an interaction effect between phonotactic probability and vocabulary on NWR performance. The general aim of the study is to investigate whether NWR, as a task of phonological storage, assesses the quality of phonological representations in children as young as 2 years of age. 557 Dutch 2-year-olds performed a NWR task containing items of varying phonotactic probability as well as a receptive vocabulary task. The results showed a moderate, significant correlation between NWR and vocabulary. Phonotactic probability had an effect on NWR performance. Further analyses showed that there was a significant interaction between phonotactic probability and vocabulary for part of the items. These results support previously reported effects of vocabulary and phonotactic probability on NWR in older, English-speaking children for a large sample of Dutch-speaking 2-year-olds, and provide evidence that NWR assesses the quality of phonological representations already in very young children.

## Introduction

Nonword repetition (NWR) has gained considerable attention in the literature on first and second language acquisition, language disorders, and dyslexia. Children’s ability to imitate unfamiliar strings of phonemes that are phonotactically legal, but lack semantics, taps many language processing abilities, and is related to vocabulary size (Gathercole and Baddeley 1989; see Gathercole 2006 for an overview). However, to date, few studies have investigated how NWR and vocabulary are associated in young children. The present study examines this relationship in a large sample of Dutch 2-year-olds. Specifically, by examining how phonotactic probability and vocabulary impact on NWR performance in these children, our aim is to investigate whether NWR assesses the quality of phonological representations already at early stages of language development.

Previous research has shown an association between NWR and vocabulary knowledge that is especially strong during early stages of language learning, that is, around the age of three or four (Gathercole et al. 1992). The few studies looking at NWR in younger children suggest that this association also holds for 2-year-olds (Gathercole and Adams 1993; Hoff et al. 2008; Stokes and Klee 2009; Zamuner 2009). To explain why NWR performance is positively correlated with children’s vocabulary knowledge, two views have been put forth.

First, the storage-based view holds that NWR assesses phonological memory capacity, or the ability to temporarily store a novel sound sequence in memory, which is important for word learning, as it mediates the storage of phonological knowledge in long-term memory (Gathercole 2006). There is a reciprocal relationship between phonological storage and vocabulary knowledge, however: Phonological storage is better for nonwords that can be supported by phonological representations in long-term memory (i.e., wordlike nonwords) than nonwords for which no or little long-term support is available. Previous studies have provided evidence that NWR taps phonological storage in children as young as 2 years of age (Gathercole and Adams 1993; Hoff et al. 2008). Gathercole and Adams (1993) found, for example, that NWR scores were closely related to two other measures of phonological memory, including a digit span task, in 2- and 3-year-olds. Hoff et al. (2008) provided evidence for NWR as a valid measure of phonological memory in English-speaking 20- to 24-month-olds.

Second, there is a representations-based view on the relationship between NWR and vocabulary that assumes that NWR ability is not the driving force behind vocabulary growth, but the result of it. Specifically, this view holds that children’s growing lexicons call for more efficient representations (Bowey 2001; Edwards et al. 2004; Masterson et al. 2005; Metsala 1999; Metsala et al. 2009; Munson et al. 2005b, a; Storkel 2002). In this view, children initially represent new words in a holistic manner, with word representations not yet capturing distinctions at the phoneme level. At later stages in lexical development, when children’s vocabularies have become larger, their word representations become more analytical at the syllable and phoneme level, enabling more efficient storage of phonological representations. At these later stages, the availability of more robust and analytical representations allows children to rearrange individual phonemes into new patterns, enabling them to repeat, for example, novel words in NWR tasks.

So, whereas in the first view, NWR is mainly seen as tapping phonological storage, driving vocabulary growth (Gathercole and Baddeley 1989), the second view holds that better NWR skill is the result of vocabulary growth, as increases in vocabulary size boost the quality of children’s phonological presentations, and thereby, NWR ability. Although researchers contend that NWR taps phonological storage as well as the quality of phonological representations in older children (Gathercole 2006; Rispens and Baker 2012), and there is evidence that it measures phonological storage in 2-year olds (Chiat and Roy 2007; Hoff et al. 2008), less is known about whether NWR assesses the quality of children’s phonological representations at very early stages of acquisition.

A robust finding in earlier work on NWR is that phonotactic probability, or the probability with which adjacent phonemes appear in actual words of a language (Jusczyk et al. 1994), impacts on NWR performance: NWR of high-phonotactic probability nonwords is typically more accurate than NWR of low-phonotactic probability nonwords (Edwards et al. 2004; Messer et al. 2010). This finding is compatible with both views on NWR. In a storage-based view, individuals ‘reconstruct’ the fuzzy or incomplete representations of stimuli that are temporarily stored in phonological memory on the basis of phonological representations in long-term memory (Gathercole et al. 2001; Thorn et al. 2002). As more support is available for high-probability items, repetition of these items is generally better than that of low-probability items. A representations-based view assumes that high-probability nonwords are repeated more accurately because the phonological processing and repetition of such items is aided by more detailed phonological representations than the processing and repetition of low-probability items.

Several studies with preschool and school-aged children have shown that the size of the phonotactic probability effect on NWR is related to vocabulary knowledge (Edwards et al. 2004; Munson et al. 2005b). Edwards et al. (2004) studied the effect of phonotactic probability on NWR in typically developing children aged 3–8 years, and found that children with large vocabularies showed a smaller phonotactic probability effect than children with smaller vocabularies. This smaller effect was due to the latter group mainly having trouble repeating the low-probability items. These results were replicated in two later studies by Munson et al. (2005b, (2005a) for both typically developing and language-impaired children between 3 and 13 years.

The results of these studies can be explained by a representations-based view on the relationship between NWR and vocabulary: If increases in vocabulary indeed boost the quality of children’s phonological representations, children with low vocabularies should especially have trouble with the repetition of low-probability items that depends strongly on the availability of high-quality phonological representations (Edwards et al. 2004; Metsala 1999; Munson et al. 2005a, b). However, if it is assumed that NWR predominantly taps phonological storage, it is not immediately clear why children with small vocabularies would perform especially poorly on low-probability items. Indeed, Gathercole et al. (1999) found that sensitivity to phonotactic frequency in serial nonword recall was equivalent in 7- and 8-year-old children with relatively high and low vocabulary knowledge for their age. On the basis of these findings, Gathercole (2006) concluded that, “the [storage-based] framework, in its current form, does not predict differential links between vocabulary knowledge and the repetition of nonwords varying in familiarity; rather, word learning ability would be expected to be associated with accuracy of repeating both low and familiarity nonwords” (p. 529). She proposes that the results of Munson et al. (2005a) may be due to a methodological flaw: In this study, performance on the high-probability nonwords showed a ceiling effect, which may have led to an underestimation of the association between vocabulary and repetition accuracy of high-probability nonwords.

For children under the age of three, few studies have looked into phonotactic probability and non-word repetition. Generally, overall effects of phonotactic probability were found (Coady and Aslin 2004; Zamuner et al. 2004; Zamuner 2009), although Coady and Aslin (2004) found that no effect of phonotactic probability surfaced when it was manipulated in single syllables rather than in the entire nonword. To date, only two studies have investigated whether there is an interaction between phonotactic probability and vocabulary knowledge in children under age three (Zamuner et al. 2004; Zamuner 2009) to find out whether NWR assesses the quality of young children’s phonological representations. In both studies, 2-year-olds performed a NWR task containing monosyllabic nonwords that were divided into high- versus low-phonotactic probability items as well as a vocabulary test. In neither of the studies, an interaction between children’s vocabulary scores and the size of the phonotactic probability effect was found. According to the authors, their null results may be due to a lack of variation in children’s vocabulary scores, obscuring any possible interactions between vocabulary and NWR performance (Zamuner et al. 2004) or to the limited set of phonemes used for constructing the stimuli (Zamuner 2009).^1^ An alternative possibility is that effects went unnoticed because in both studies the analyses of NWR accuracy were restricted to the repetition of segments in specific positions (onset or coda) rather than all segments in a nonword.

Summarizing, previous studies have shown that NWR correlates positively with vocabulary, already at 2 years of age (Gathercole and Adams 1993; Hoff et al. 2008; Chiat and Roy 2007; Zamuner 2009), which is compatible with both the storage-based and representations-based view on NWR. Furthermore, earlier work has shown that phonotactic probability affects NWR in 2-year-olds (Zamuner et al. 2004; Zamuner 2009), even though, at this young age, sensitivity to phonotactic probability may not yet be entirely in place (Coady and Aslin 2004). Finally, previous studies show that vocabulary knowledge predicts the size of the phonotactic probability effect in preschool and school-aged children such that children with low vocabularies show a larger probability effect than children with large vocabularies, as the former perform poorly on in particular low-probability items (Edwards et al. 2004; Munson et al. 2005a). However, for younger children, previous studies have failed to show interaction effects between phonotactic probability and vocabulary knowledge on NWR (Zamuner et al. 2004; Zamuner 2009). One possible explanation of these null findings concerns the type of stimuli used in previous work.

In this study, we examine data from a large sample of Dutch-speaking 2-year-olds in order to shed more light on the relationships between NWR, phonotactic probability and vocabulary in children this young. Our general aim is to see whether, apart from phonological storage, NWR assesses the quality of children’s phonological representations at 2 years of age. We target three questions. First, we ask whether NWR and vocabulary are correlated in this sample, to follow up on previous studies finding such a relationship in children under age three (Chiat and Roy 2007; Gathercole and Adams 1993; Hoff et al. 2008; Stokes and Klee 2009; Zamuner 2009). Second, we examine whether children are influenced by phonotactic probability in NWR performance, investigating a much larger sample than in previous studies and analyzing all segments in the nonwords rather than only segments in onset or coda position. Finally, if we find an effect of phonotactic probability, we assess whether this effect is related to children’s vocabulary knowledge. Specifically, we investigate if children with small vocabularies will have problems repeating low-probability items in particular when compared to children with larger vocabularies, as has been found for school-aged children (Edwards et al. 2004; Munson et al. 2005b, a). If this is the case, this would present evidence that, besides phonological storage, NWR taps the quality of phonological representations in children as young as 2 years.

## Method

### Participants

Participants were 557 Dutch monolingual 2-year-old children with a mean age of 27 months ($SD = 3$, range 23–36, 262 boys) who had completed a NWR and vocabulary task. As for socio-economic status (SES) 361 children came from high SES backgrounds (65 %), defined as having one or both parents who had completed higher education. The remainder of the children (35 %) came from families in which parents had completed vocational or intermediate education as their highest level. The children investigated in this study are a subset of 1044 Dutch 2-year-old children out of a larger group who participated in a large-scale longitudinal cohort study (i.e., the pre-COOL study) examining language, executive function, motor and social-emotional development in children between 2 and 5 years of age (Mulder et al. 2014).^2^ Participants in this study had been recruited from nurseries, playgroups and directly from families via a mailing sent out to parents of children born between 1st of April 2008 and 1st of November 2008 asking for their participation. The data reported on in the present study were collected during the first wave of data collection, when children were 2 years old. On the basis of a letter informing parents about the research project, parents permitted participation through informed consent (children tested at home) or declined through opting out (children tested at day care centers).

The current subsample consisted of children whose parents had returned a questionnaire that contained biographical questions about the language(s) spoken at home, hearing/vision, and family characteristics such as SES (i.e., 83 % of all parents of Dutch monolingual children with scores on the relevant tasks). After exclusion of children with neurological problems ($N = 1$), hearing impairment ($N = 2$), Down syndrome ($N = 2$), and children who did not complete the vocabulary task ($N = 48$), the sample consisted of 984 children. Out of these, 904 children attempted to repeat at least one nonword (92 %), similarly to findings in other studies on NWR in young children (Chiat and Roy 2007; Hoff et al. 2008; Zamuner et al. 2004). Following other studies (Gathercole and Adams 1993; Zamuner 2009), only children who responded to all NWR test items were included, which was 62 % of the responders sample ($N = 557$).

Previous studies have shown that children who are willing to repeat the items in a NWR task may differ from children who do not respond in, amongst others, vocabulary knowledge (Hoff et al. 2008; but see Chiat and Roy 2007). Indeed, in our sample, children who repeated one or more NWR items obtained significantly higher receptive vocabulary scores than children who did not respond to any of the items (51 vs. 67 % correct, $F(1{,}983) = 53.55, p <.001, \eta ^{2}_{p} = .05$). Therefore, after having presented the results for the children who responded to the full task, we will repeat our analyses on a broader sample of children who did not respond to all items, to see whether this yields the same results.

### Tasks

#### Nonword Repetition

A NWR task was developed for the purposes of the current study, because no tasks for Dutch-speaking toddlers were available in which phonotactic probability had been manipulated across segments in each nonword (but see Zamuner 2009) for a task targeting the influence of phonotactic probability on Dutch children’s repetition of specific (word-initial or word-final segments). The task included twelve items, half of which were monosyllabic and half bisyllabic. All items were composed of phonemes that are known to be acquired early by Dutch children, to avoid articulation difficulties as much as possible (Beers 1995; Fikkert 1994). Furthermore, they did not contain consonant clusters and contained a maximum of one diphthong per item. Stress was always on the first syllable in the bisyllabic items, following the most typical stress pattern in Dutch (Daelemans et al. 1994).

The items were manipulated for phonotactic probability, by calculating log-frequency counts of the CELEX database (Baayen et al. 1995) with the help of the software Phonotactools (Adriaans 2006). Since previous research on Dutch 2-year-old children has shown that children are particularly sensitive to the first phoneme in nonwords (Zamuner 2009), items were constructed in pairs such that for each high-probability item there was a low-probability counterpart of the same length starting with the same phoneme (e.g., ‘loen’ vs. ‘luup’).

Table 1 lists the mean biphone transitional log frequencies and wordlikeness ratings for the mono- and bisyllabic items of high- versus low-probability, as well as for each individual item. The high-probability nonwords had significantly higher biphone transitional frequencies than the low-probability nonwords ($F(1{,}11) = 28.42, p < .001, \eta ^{2}_{p} = .74$). Item length as a covariate was not significant, indicating that the differences in phonotactic probability did not differ for the mono- and bisyllabic items ($p > .1$). In order to assess whether the distinction between the low- and high-probability items was perceived by native speakers, 15 adults were asked to rate the items for wordlikeness on a scale that ranged from 1 (does not sound like a Dutch word at all) to 10 (sounds very much like a Dutch word). As can be seen from Table 1, low-probability items obtained significantly lower ratings than high-probability items ($F(1{,}14) = 32.80, p < .001, \eta ^{2}_{p} = .70$). The monosyllabic items were judged as being more wordlike overall than the bisyllabic items ($F(1{,}14) = 7.56, p = .016, \eta ^{2}_{p} = .35$). No interaction effects were found ($p\hbox {s} > .1$). A paired *t*-test showed that the differences in wordlikeness ratings of high- versus low-probability items held for the monosyllabic items ($t(1,14) = 18.26, p = .001, \eta ^{2}_{p} = .57$) and bisyllabic ones ($t(1,14) = 9.02, p < .001, \eta ^{2}_{p} = .39$). The task had good internal consistency as indicated by Cronbach’s alpha ($\alpha = .83$).

**Table 1:** Biphone log frequencies for high- and low-phonotactic probability (PP) items and wordlikeness ratings by Dutch native speakers

Item		PP	Wordlikeness rating
*All items*		−1.39	5.39
Monosyllabic high-PP		−1.01	6.51
Monosyllabic low-PP		−1.59	5.18
Bisyllabic high-PP		−1.04	5.36
Bisyllabic low-PP		−1.54	4.53
*Monosyllabic*			
High-PP			
jaat	/ja:t/	−0.66	6.80
peek	/pe:k	−1.18	6.73
loen	/lun/	−1.19	6.00
Low-PP			
jiek	/ji:k/	−1.62	4.13
peun	/pøn/	−1.56	6.47
luup	/ly:p/	−1.58	4.93
*Bisyllabic*			
High-PP			
holin	/’ho:lIn/	−1.07	5.33
natep	/’na:t$\upvarepsilon$p/	−0.79	4.93
kepon	/’ke:pOn/	−1.25	5.80
Low-PP			
hiemup	/’hi:mʏp/	−1.52	3.67
nuipok	/’nœypOk/	−1.62	3.93
keupun	/’køpʏn/	−1.48	6.00$^{\mathrm{a}}$

$^{\mathrm{a}}$ The relatively high rating for the item ‘keupun’ may be due to the syllable ‘keu’ being a real word in Dutch (meaning a cue used in billiards). However, this word is very low-frequent and likely to be unknown in young children

All items were pre-recorded in a soundproof room by a female speech therapist using a high pitch voice that is typical of child-directed speech. The recordings were then embedded in the following procedure to keep children engaged in the task: Children watched short video clips in which a novel object appeared from a picture of a box. At the same time, they heard a prerecorded sentence labeling the object that encouraged them to repeat the nonword: *‘Look, a [jaat]! Say [jaat]!* 3 The purpose of playing movie clips and pre-recorded speech on a laptop was to keep children engaged in the ‘game’, while at the same time, ensuring the uniformity of input in terms of rate, pitch, volume, and other phonetic and auditory features that may otherwise vary across and within speakers. Such uniformity of input seemed especially important in a study of this scale in which different research assistants administered the task. Furthermore, this approach circumvents the need to cover the mouth to avoid visual cues, as has been done in some previous studies.

Items were presented in a fixed, pseudo-randomized order: No more than two items of the same type were presented after one another. If children did not repeat an item, experimenters used the prompt sentence ‘What’s that?’ to elicit a response from the child without repeating the target item. If children still did not repeat the item, the recording was played again, with a maximum of two repetitions (similar to Hoff et al. 2008; Roy and Chiat 2004).^4^ Two practice trials were presented prior to the test phase to familiarize children with the task, and short breaks were allowed if children lost attention or did not want to proceed.

#### Receptive Vocabulary

The Dutch version of the Peabody Picture Vocabulary Test (PPVT) was used to assess receptive vocabulary (PPVT-III-NL, Dunn and Dunn 2005). In this task, children choose one out of four picture drawings after an orally presented word. Whereas this task is usually performed as a paper-and-pencil task, stimuli presentation in the current study was controlled by the experimental software E-Prime 2.0 (Psychology Software Tools, Pittsburgh, PA), and administered through a laptop to facilitate administration and scoring. Since some children were younger than 27 months, the starting age for this test, we chose to present each child with the same test items rather than apply the standard adaptive protocol. Also, due to time constraints, the test was shortened from twelve to eight items per set, so that each child was presented with the same 24 test items in total. A pilot study with 111 children ($M = 28$ months, $SD = 3$, range 23–37) established that the items that were removed did not differentiate well among children, because they were either very easy or very difficult for 2-year-olds (i.e., mean scores on these items were either below 30 % or above 70 % correct). Because of the adjustments made to the test as well as the young age of some of the participants, raw scores rather than standard scores were used. Inspection of the scores showed that this was justified, as even the youngest children in the sample showed considerable variation in scores and performed well above chance. Internal consistency of the task was high ($\alpha = .88$).

### Procedure

Children were tested individually by trained research assistants in a quiet room at their day care center or at home. The tasks were administered in a fixed order as part of a larger test battery (Mulder et al. 2014). In all sessions, the receptive vocabulary task was presented prior to the NWR task. Test sessions lasted for approximately 45 min. At the end of the session, children received a small gift.

### Scoring

Children’s responses on the NWR task were scored as correct if they contained all phonemic segments of the target in the correct order with no additional phonemes (Chiat and Roy 2007; Gathercole et al. 1994). Responses were scored online by trained assistants. Scoring options were ‘correct’, ‘incorrect’, ‘unclear’, and ‘no response’. The option ‘unclear’ was used if children’s responses could not be categorized as either correct or incorrect by the assistant (2 % of all responses), and were left out of the analyses. To minimize inter- and intra-individual differences in online scoring, all assistants had attended an extensive training and submitted a video of a practice session with a 2-year-old child to the principal investigators prior to testing, on which they were given elaborate feedback. If deemed necessary, they were requested to submit a second video of another practice session. These videos indicated that disagreement among scorers was extremely rare, presumably due to all items being short and phonologically simple. Indeed, in a separate study with the same NWR task presented to 45 Dutch monolingual 2-year-olds ($M = 30$ months, $SD = 3$, range 24–35) in which we used phonemic transcriptions, we found that assistants’ online correct/incorrect scores correlated highly with these same assistants’ offline scores on the basis of phonemic transcriptions (*r* = .84) as well as with those of a second rater ($r = .82$).

Although a substantial number of studies have used more fine-grained scores such as phoneme percentage correct, several authors have found no differences between more and less subtle scoring methods (Roy and Chiat 2004; Thal et al. 2005). Roy and Chiat (2004), for example, compared three measures in a study on 2- to 4-year-old children: (i) whole items correct, (ii) number of phonemes correct making allowances for articulatory and sociolinguistic variations, and (iii) number of phonemes correct with no allowances. Their results showed that the use of the more precise scores did not make a difference to their results and did not prove more informative than the global score of whole items correct. In our smaller study with 45 children, we found a strong correlation between mean scores based on whole items correct and mean scores based on phoneme percentage correct ($r(45) = .85, p < .001$). Also, this study showed very similar correlations between NWR and receptive vocabulary, assessed with the same receptive vocabulary test, for scores based on whole items correct and phoneme percentage correct ($r(45) = .29$ and $r(45) = .31$).

### Analyses

To answer our question whether vocabulary and NWR would be correlated, we used a Pearson’s correlation analysis, as vocabulary and NWR scores were normally distributed in our sample. As for our second question on effects of phonotactic probability, we ran a repeated-measures ANOVA with item length and phonotactic probability as within-subject factors, and children’s mean NWR scores as the dependent variable. To answer our final question about a possible interaction between phonotactic probability and vocabulary, linear logistic mixed-effect models were run in the statistical program (R Development Core Team 2008). Linear mixed-effect models or ‘mixed-effect models’ can be used for testing the simultaneous effects of both continuous and categorical variables on a dependent variable. In our model, vocabulary and phonotactic probability were continuous variables and item length was a categorical variable (one vs. two syllables). Children’s repetition accuracy (i.e., correct/incorrect scores on each item) was entered as the dependent variable. As this variable is categorical (i.e., correct/incorrect), we used linear logistic models.

An advantage of mixed-effect models is that, in such models, participants and items can be modeled as random-effect factors, which allows for the conclusion that the model based on the current sample of participants and items can be generalized to the larger set (population) of which the sample of subjects and items was drawn. In the present analyses, phonotactic probability was treated as a continuous factor rather than a dichotomous one (‘high’ vs. ‘low’), thus reflecting the nature of the stimuli more closely (see Janse and Newman 2013 for a similar analysis on effects of phonotactic probability on NWR in adults). The reason for this was twofold: First, a gradient approach to phonotactic probability resembles real life input more adequately than a dichotomous classification, and secondly, there was considerable variation within the set of high- and low-probability items in terms of probability values (see Table 2 above) that could not be dealt with if phonotactic probability was treated as a categorical variable.

## Results

Mean percentages correct and standard deviations for the NWR and vocabulary tasks are presented in Table 2.

**Table 2:** Descriptive statistics for PPVT and NWR (means indicate % accurate responses)

	Mean	Range	SD	N
*PPVT*	70.75	12.50–100	16.24	557
*NWR*				
All items	43.73	0–100	27.23	557
*Monosyllabic*				
High-probability	59.54	0–100	34.15	557
Low-probability	46.92	0–100	35.38	557
*Bisyllabic*				
High-probability	37.16	0–100	34.82	557
Low-probability	31.30	0–100	32.79	557

These results show that there was substantial variation in performance on the NWR task across children, as indicated by the large standard deviations. One possible explanation of this variation may be due to differences in age. However, even though the correlation between age and NWR performance was significant, it was rather weak ($r(557) = .19, p < .001$), and much lower than the correlation between age and vocabulary scores ($r(557) = .37, p < .001$). Thus, differences in age did not strongly associate with differences in NWR ability. Another explanation of the high variability in NWR scores is the early stage of development the 2-year-olds are in, which is likely to coincide with high inter-child variation in language abilities.

### Vocabulary and NWR

As for our first question on the association between NWR and vocabulary, we found a positive, moderate and significant partial correlation between vocabulary scores and NWR performance ($r(557) = .30, p < .001$), after controlling for age. This result is in line with partial correlations reported in earlier studies (Chiat and Roy 2007; Gathercole and Adams 1993).

### Phonotactic Probability and NWR

Our second question was whether 2-year-olds would show sensitivity to phonotactic probability in NWR performance, that is, whether they were able to repeat high-probability nonwords more accurately than low-probability nonwords. NWR results for the high- and low-probability nonwords are presented in Table 2 for the one- and two-syllable nonwords separately. A repeated measures ANOVA with ‘length’ and ‘phonotactic probability’ as within-subject factors showed a main effect of phonotactic probability, with children repeating high-probability nonwords significantly more accurately than low-probability nonwords ($F(1{,}556) = 87.47, p < .001, \eta ^{2}_{p} = .14$), a main effect of length, such that monosyllabic items were repeated significantly more accurately than bisyllabic items ($F(1{,}556) = 319.17, p < .001, \eta ^{2}_{p} = .37$), and a small interaction effect between length and phonotactic probability ($F(1{,}556) = 11.28, p = .001, \eta ^{2}_{p} = .02$). A paired-samples *t* test indicated that there was a phonotactic probability effect for the monosyllabic items and bisyllabic items, but that the effect was larger for the monosyllabic ($t(1,556) = 8.50, p < .001, \eta ^{2}_{p} = .12$) than for the bisyllabic items ($t(1,556) = 4.40, p < .001, \eta ^{2}_{p} = .03$).

### Vocabulary and the Effect of Phonotactic Probability

Our final aim was to test for effects of vocabulary and phonotactic probability on children’s NWR accuracy, and in particular, for possible interaction effects between vocabulary and phonotactic probability. A linear logistic mixed-effect model with item length, vocabulary, and phonotactic probability as the independent variables and repetition accuracy of each item (i.e., correct/incorrect) as the dependent variable showed a main effect of vocabulary, but no main effects of length or phonotactic probability. There were three interaction effects: a two-way interaction effect between vocabulary and item length, a two-way interaction between vocabulary and phonotactic probability, and a three-way interaction between vocabulary, item length and phonotactic probability. Results are reported in Table 3.

**Table 3:** Model estimates, standard error (SE), Z- and *p*-values of mixed-effect regression model

	Coefficient	*SE*	*Z*	*p*
(Intercept)	−2.65	2.49	−1.07	.286
Length	1.33	1.65	0.80	.422
Vocabulary	0.11	0.03	4.15	<.001
PP	−0.96	1.86	−0.52	.604
Length $\times$ vocabulary	−0.06	0.02	−3.39	<.001
Vocabulary $\times$ PP	−0.05	0.02	−2.76	.006
Length $\times$ PP	−1.85	1.25	−1.48	.140
Length $\times$ vocabulary $\times$ PP	0.04	0.01	3.39	<.001

*Note*. As a higher log frequency represents a lower phonotactic probability, effects involving phonotactic probability are negative

To examine these interaction effects more closely, we ran separate logistic mixed-effect models for the mono- and bisyllabic items. In these models, we tested for effects of vocabulary, phonotactic probability and vocabulary*phonotactic probability on repetition accuracy. Results are reported in Table 4.

**Table 4:** Model estimates, standard error (SE), Z- and *p*-values of mixed-effect regression models for mono- and bisyllabic items

	Coefficient	*SE*	*Z*	*p*
*Model for monosyllabic items*				
(Intercept)	−1.35	0.96	−1.40	.163
Vocabulary	0.05	0.01	4.37	<.001
PP	−0.81	0.70	−1.16	.247
Vocabulary $\times$ PP	−0.01	0.01	−1.20	.228
*Model for bisyllabic items*				
(Intercept)	−0.01	1.41	−0.01	.996
Vocabulary	−0.01	0.01	−0.57	.567
PP	−2.80	1.06	−2.65	.008
Vocabulary $\times$ PP	0.03	0.01	3.54	<.001

*Note*. As a higher log frequency represents a lower phonotactic probability, effects of phonotactic probability are negative

These results show different effects for the mono- and bisyllabic items. For the monosyllabic items, there is a main effect of vocabulary ($p < .001$), but no effect of phonotactic probability. For the bisyllabic items, the opposite pattern is found: There is no effect of vocabulary, but phonotactic probability does have an effect ($p < .01$). Moreover, for these items, there is an interaction effect between vocabulary and phonotactic probability ($p < .001$).

The absence of an effect of phonotactic probability for the monosyllabic items is surprising. One possibility is that since the effect of vocabulary is strong and vocabulary and phonotactic probability are correlated, entering vocabulary and phonotactic probability together in the model does not leave much room for phonotactic probability as a predictor. Indeed, entering phonotactic probability as the sole predictor in the model for monosyllabic items yields a strong effect of this factor ($b = -1.47, SE = .43, z = -3.41, p < .001$). Likewise, entering vocabulary as the sole predictor in the model for bisyllabic items yields a strong effect of vocabulary ($b = .04, {SE} = .00, z = 7.51, p < .001$). So, it is only when both factors are entered together that clear differences between the two types of items arise.

**Fig. 1:**
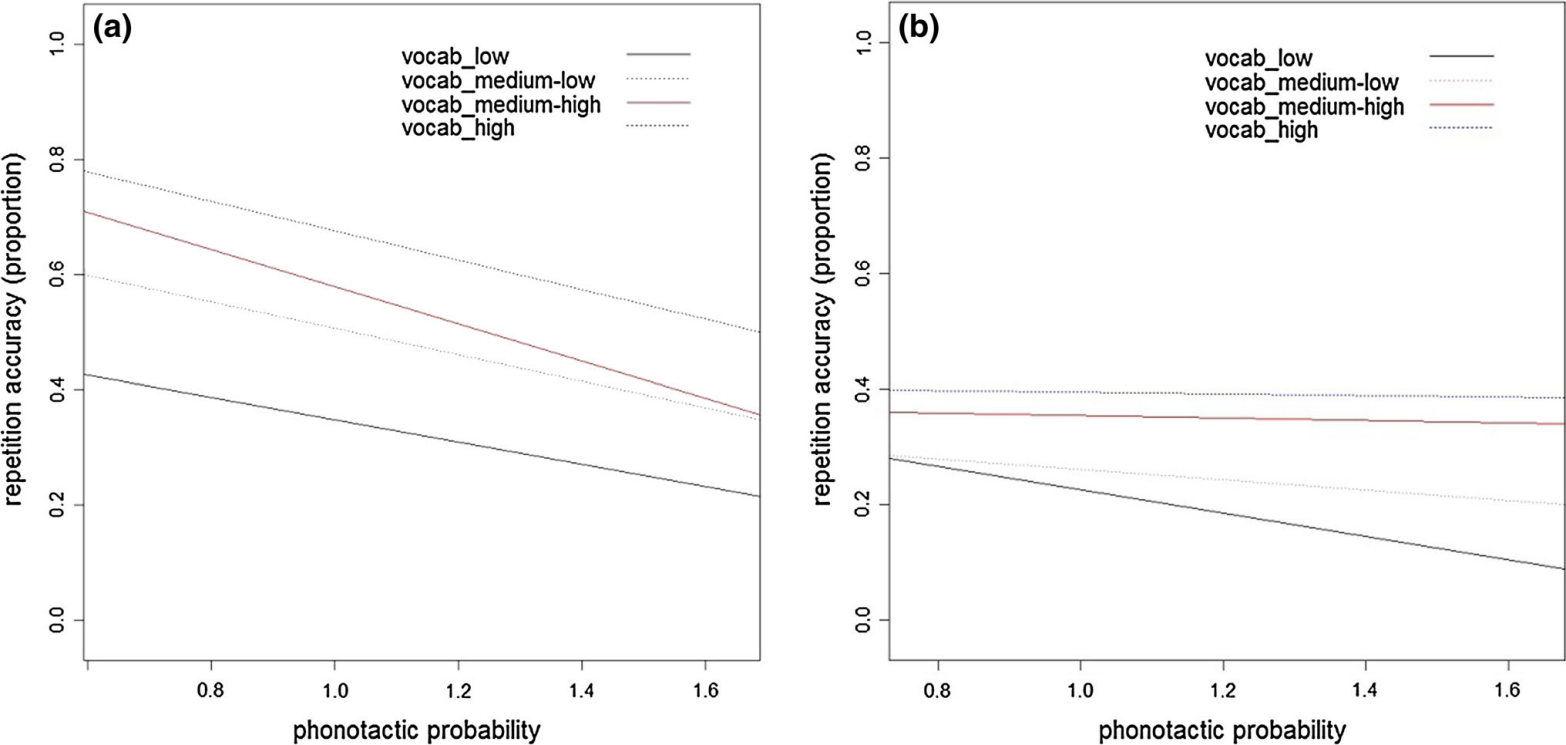
Proportion correct for monosyllabic items (**a**) and bisyllabic items (**b**) in relation to phonotactic probability for children with low, medium-low, medium-high, and high vocabulary scores

Interaction effects between two continuous variables are difficult to interpret. In order to visualize the interaction, we split the children into four different vocabulary groups, based on the interquartile range in vocabulary scores. Figure 1a, b represent the results graphically, showing children’s NWR accuracy as a function of phonotactic probability for the four vocabulary groups separately. Separate graphs are provided for children’s performance on the monosyllabic items (Fig. 1a) and bisyllabic items (Fig. 1b). Figure 1a shows a clear trend with scores becoming lower with decreasing phonotactic probability for all groups, even though the effect of phonotactic probability was not significant. Figure 1b shows that the vocabulary*phonotactic probability interaction found for the bisyllabic items is due to a stronger influence of phonotactic probability in the low vocabulary group, whose performance is particularly poor on the low-probability items. This figure also shows that phonotactic probability does not seem of influence in the groups that obtained high vocabulary scores.

### Applying a Less Stringent Criterion: Same Results for a Larger Sample?

The results reported so far were based on children who had responded to all items in the NWR task, and might present an overestimate of 2-year-olds’ NWR skill. In fact, as we reported above, children who responded to all items in the NWR task significantly outperformed the non-responders on the receptive vocabulary task. In order to assess whether the same patterns were obtained if a less stringent inclusion criterion was used, we repeated our analyses on the sample of children who responded to at least three out of six high-probability items and three out of six low-probability items. This criterion yielded a sample of 796 children with a mean age of 27 months ($SD = 3$, range 22–36) of which 384 were boys (48 %).

First, a positive moderate correlation was found between NWR performance and vocabulary ($r(796) = .29, p < .001$), a finding in line with the correlation found above. Second, a repeated-measures ANOVA showed very similar effects as the above analysis: a main effect of phonotactic probability ($F(1{,}795) = 119.98, p < .001, \eta ^{2}_{p} = .13$), a main effect of length ($F(1{,}795) = 441.59, p < .001, \eta ^{2}_{p} = .36$), and a small interaction effect between phonotactic probability and length ($F(1{,}795) = 11.07, p = .001, \eta ^{2}_{p} = .01$). Linear logistic mixed-effect models with items and subjects as random factors and length, vocabulary and phonological probability as fixed factors showed the same pattern of results as for the restricted sample. In the model based on all items, there was a main effect of vocabulary ($b = .08, SE = .02, z = 3.97, p < .001$), as well as significant interactions between length and vocabulary ($b = -.04, SE = .01, z = -2.76, p = .006$), vocabulary and phonotactic probability ($b = -.03, SE = .01, z = -2.22, p = .026$), and between length, vocabulary and phonotactic probability ($b = .03, SE = .01, z = 2.76, p = .006$). Also, in the models that were run for the mono- and bisyllabic items separately, the same effects were found as for the smaller sample. That is, for the monosyllabic items, there was an effect of vocabulary ($b = .04, SE = .01, z = 4.86, p < .001$), but there was no effect of phonotactic probability and no interaction between vocabulary and phonotactic probability ($p\hbox {s} > .1$). For the bisyllabic items, there was no effect of vocabulary ($b = .00, SE = .01, z = .36, p = .716$), but there was a main effect of phonotactic probability ($b = -1.49, SE = .74, z = -2.01, p = .045$) as well as an interaction effect between vocabulary and phonotactic probability ($b = .02, SE = .01, z = 2.45, p = .014$). These results closely match those for the more restricted sample that were reported above.

## Discussion

This study investigated the relationships between NWR, vocabulary, and phonotactic probability in a large sample of Dutch-speaking 2-year-olds. Three questions were addressed. The first question was whether NWR and vocabulary correlated in these children, the second whether NWR performance of 2-year-olds was influenced by phonotactic probability, and the final question looked into a possible interaction between phonotactic probability and vocabulary on NWR ability. A positive answer to the last question would suggest that NWR assesses the quality of phonological representations in children this young, in line with previous findings for older children (Metsala 1999; Munson et al. 2005a; Rispens and Baker 2012).

Our results showed a significant, moderate correlation between NWR and receptive vocabulary in Dutch children, in line with previous findings on English-speaking children (Baddeley et al. 1998; Bowey 2001; Gathercole et al. 1994; Jarrold et al. 2004; Munson et al. 2005a), including studies on 2-year-olds (Chiat and Roy 2007; Gathercole and Adams 1993; Hoff et al. 2008; Stokes and Klee 2009; Zamuner 2009). These findings confirm that the two abilities are associated for a much larger sample than investigated in previous studies.

For the second question, we found a main effect of phonotactic probability in an ANOVA treating phonotactic probability as a categorical variable as well as in a linear logistic mixed-effect model taking into account the continuous character of the items’ phonotactic probability values. In the latter analysis, such an effect emerged for both mono- and bisyllabic items when vocabulary was not entered in the model. The presence of a phonotactic probability effect on NWR performance sits well with studies that have reported benefits of phonotactic probability in repetition of novel items with school-aged children (e.g., Edwards et al. 2004; Munson et al. 2005b, a). This finding also aligns with the studies of Zamuner (2009), Zamuner et al. (2004) and Coady and Aslin (2004), who reported probability effects for children aged 2 years.

Finally, our results showed an interaction between vocabulary and phonotactic probability, as has been found for older children (Edwards et al. 2004; Munson et al. 2005a). This interaction was, however, dependent on item length, as it was only found for the bisyllabic, and not for the monosyllabic items. The interaction showed that children with low receptive vocabulary scores performed especially poorly on the low-probability items. This is in line with previous results on older, English-speaking children (Edwards et al. 2004; Munson et al. 2005a), and suggests that NWR taps phonological representations that become more elaborate with increasing vocabulary knowledge, already in children as young as 2 years of age. Thus, the current findings suggest that switches from more global to analytical phonological representations take place early in life, and can be detected with NWR in children under age three.

Importantly, the interaction effect between vocabulary and phonotactic probability only held for the bisyllabic items in our task, and only children with low or medium vocabulary scores showed such an effect. The first finding is similar to results of Jones et al. (2007) who found that English 2- to 5-year-old children showed a stronger effect of phonotactic probability in three- and one-syllable nonwords than in two-syllable nonwords. The authors do not explain this finding, since the focus of their study is on a computational model of vocabulary acquisition rather than on NWR data elicited from children. One possible explanation of such interactions is that item length correlates with other factors known to influence NWR, such as neighborhood density and articulation difficulty that may be differentially related to phonotactic probability. In the current study, we could not look into the possible effects of such factors, as the large-scale character of our study did not allow for such an in-depth investigation. Thus, it remains for further research to examine if—and if so, why—effects of phonotactic probability interact with specific item characteristics such as item length.

One speculative explanation of why no interaction was found for the monosyllabic items in the current study is that the repetition of such items was influenced by these items being more similar to real words than the bisyllabic items, as indicated by higher wordlikeness ratings. Our validation study supports this idea as it showed that children regularly repeated monosyllabic nonwords as phonologically similar, real words (e.g., loen > bloem ‘flower’, jiek > ziek ‘ill’), whereas such errors were virtually absent for bisyllabic nonwords. In a similar vein, one may wonder whether the interaction between vocabulary and phonotactic probability found for the bisyllabic nonwords could also be due to such a direct encoding strategy: The bisyllabic nonwords of low-probability generally had lower wordlikeness ratings than the bisyllabic nonwords of high-probability (even though not significantly higher). This leaves open the possibility that our finding that children with large vocabularies especially had trouble repeating low-probability items was due to the fact that these nonwords could not be easily bootstrapped from known, existing words by these children. Future work could include an additional measure of phonological representations (e.g., mispronunciation detection tasks) next to vocabulary and NWR tasks, to enable an investigation of the exact role vocabulary plays in NWR. Moreover, older children might be tested, who, due to their larger lexicons, are predicted to be able to use direct encoding for the low-probability nonwords as well. However, in this case, it would be important to include subtler response measures, such as fluency measures and perhaps even add time pressure to the task, to avoid ceiling effects.

In relation to this last issue of response measurement, an alternative interpretation of our finding that an interaction only emerged for the bisyllabic items is that the current response measure (correct or incorrect) was sensitive enough to reveal interaction effects in the monosyllabic items. Edwards et al. (2004) found that 3- to 8-year-old children produced the same target phonemes less fluently in low- versus high-probability nonwords. This raises the question if, in the current study, different results had been obtained if fluency measures had been used next to accuracy scores. Fluency, in turn, is related to articulation. Previous work shows that children’s motor practices are related to vocabulary size (e.g., Schwarz et al. 2006), novel word learning (e.g., Schwartz and Leonard 1982), and specifically to nonword repetition (Edwards et al. 2004; Keren-Portnoy et al. 2010; see Stoel-Gammon 2011 for an overview). Thus, motor practice is important for NWR, either directly, or through other skills related to NWR, such as vocabulary knowledge. In our study, only a receptive measure of vocabulary was included. There is some evidence that correlations with nonword repetition are larger for expressive vocabulary than for receptive vocabulary (Hoff et al. 2008), raising the question to what extent NWR is related to children’s articulation skills (cf. Keren-Portnoy et al. 2010). However, Hoff et al. (2008) found that the significant correlation between NWR and vocabulary in their sample of 2-year-olds remained if they removed the shared variance between NWR and children’s real word repetition, suggesting that articulation does not play a key role.

The present findings were obtained from a large sample of Dutch children. Findings were highly similar for a sample of children who completed the NWR task and a sample of children who repeated at least half of the stimuli, suggesting that our results were not limited to a specific subgroup. Previous studies on NWR have often been based on children who responded to the entire task only, and this sampling bias may have affected the results. In the current study, for instance, vocabulary scores were significantly higher in the more restricted sample than in the broader sample. Yet, effects of vocabulary and phonotactic probability were very similar regardless of which sample was looked at, suggesting that these were robust.

Taken together, our results show that NWR does not only tap phonological storage but also the quality of phonological representations in children as young as 2 years of age (Gathercole 2006; Metsala et al. 2009). These findings match findings from Rispens and Baker (2012) who assessed NWR in 5- and 7-year-old Dutch children and found that measures of storage (digit span) as well as measures of representation (mismatch detection) predicted NWR outcomes (see also Chiat 2006).

The question of whether NWR drives vocabulary learning or vice versa calls for longitudinal data, allowing for growth analyses of both skills over time. We are currently analyzing NWR and vocabulary data of the current sample at older ages to see how the relationships between NWR, vocabulary and phonotactic probability unfold over time. The present results are a first step towards examining the relationships between vocabulary and NWR at an early phase of language development, in children as young as 2 years of age.

## Footnotes

1 In Zamuner (2009), children were tested on how they repeated the same phonemes in different phonotactic environments (e.g., /p/ in /pim/ vs. /p/ in /pat/), whereas Munson et al. (2005a) tested how children produced different segmental sequences (e.g., /pw/ in /pwageb/ vs. /tw/ in /tweket/).

2 In the longitudinal national cohort study pre-COOL, a large sample of over 2500 children is being followed during the preschool years, from age 2 to 5 years. The main aim of this study is to investigate effects of early child care and education on children’s linguistic, general cognitive and social-emotional development.

3 Previous studies on NWR in young children have also used labeling procedures in which children were asked to repeat a novel label for a picture or object (cf. Bree et al. 2006; Hoff et al. 2008; Zamuner et al. 2004).

4 A possible concern is that the repetition of stimuli may have led to increased NWR performance. However, it is highly unlikely that repetitions of the stimuli had an impact on our results. In a separate study with 45 Dutch 2-year-olds performing the same NWR task, we found that repetitions were highly infrequent, occurring in only 3 % of the trials.
